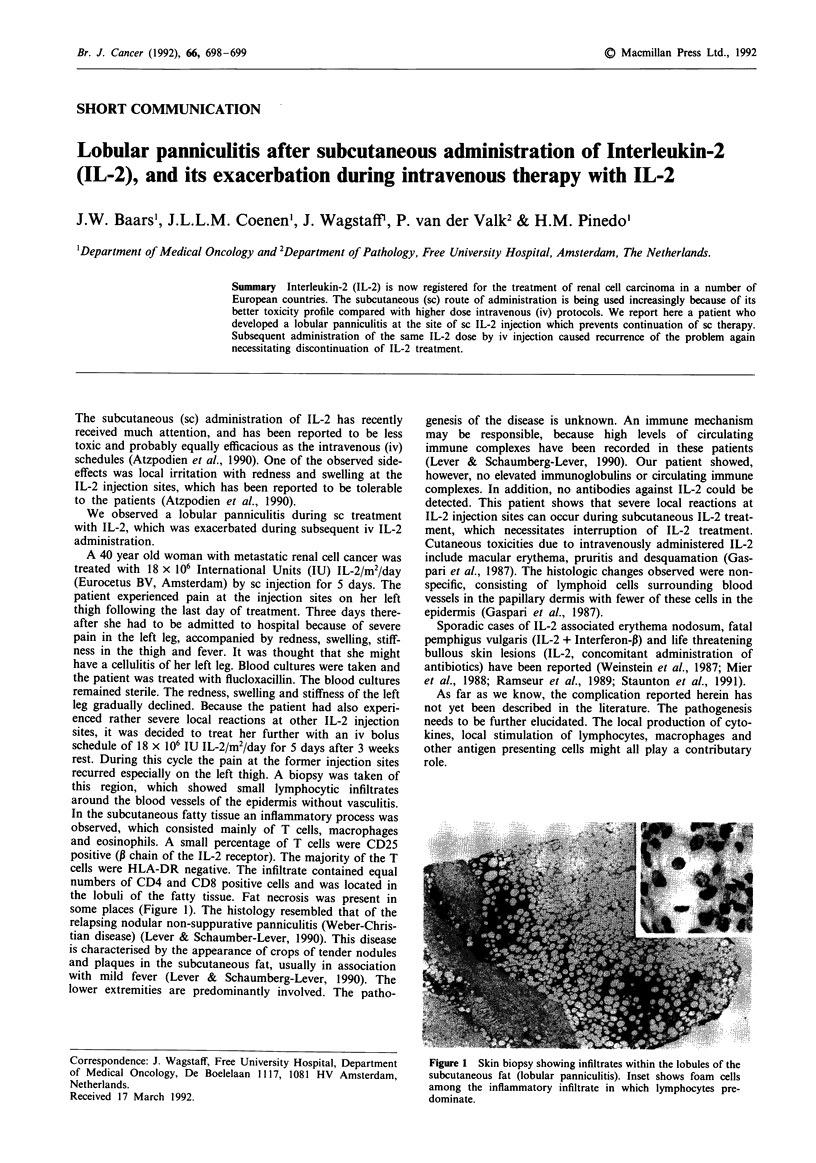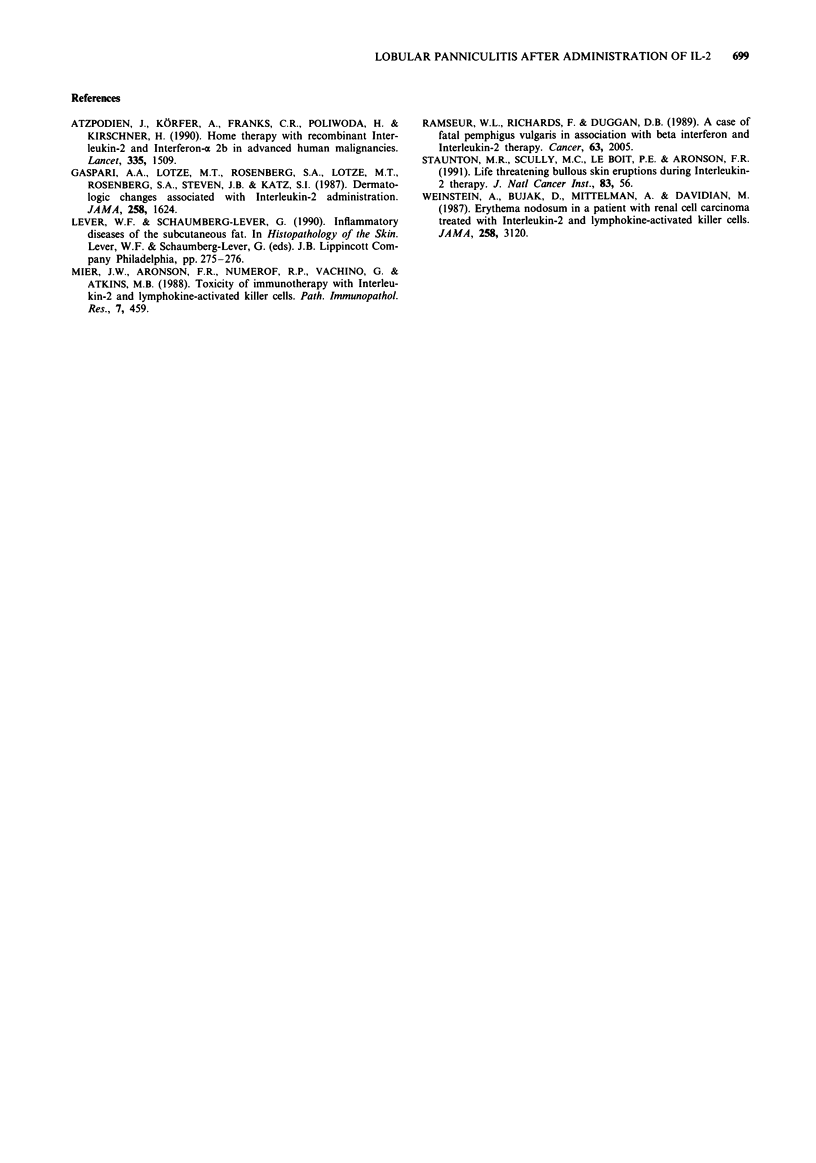# Lobular panniculitis after subcutaneous administration of interleukin-2 (IL-2), and its exacerbation during intravenous therapy with IL-2.

**DOI:** 10.1038/bjc.1992.340

**Published:** 1992-10

**Authors:** J. W. Baars, J. L. Coenen, J. Wagstaff, P. van der Valk, H. M. Pinedo

**Affiliations:** Department of Medical Oncology, Free University Hospital, Amsterdam, The Netherlands.

## Abstract

**Images:**


Br. J. Cancer (1992), 66, 698–699 © Macmillan Press Ltd., 1992

SHORT COMMUNICATION

### Lobular panniculitis after subcutaneous administration of Interleukin-2 (IL-2), and its exacerbation during intravenous therapy with IL-2

J.W. Baars[1], J.L.L.M. Coenen[1], J. Wagstaff[1], P. van der Valk[2] & H.M. Pinedo[1]

[1]*Department of Medical Oncology and* [2]*Department of Pathology, Free University Hospital, Amsterdam, The Netherlands.*

**Summary** Interleukin-2 (IL-2) is now registered for the treatment of renal cell carcinoma in a number of European countries. The subcutaneous (sc) route of administration is being used increasingly because of its better toxicity profile compared with higher dose intravenous (iv) protocols. We report here a patient who developed a lobular panniculitis at the site of sc IL-2 injection which prevents continuation of sc therapy. Subsequent administration of the same IL-2 dose by iv injection caused recurrence of the problem again necessitating discontinuation of IL-2 treatment.

The subcutaneous (sc) administration of IL-2 has recently received much attention, and has been reported to be less toxic and probably equally efficacious as the intravenous (iv) schedules (Atzpodien *et al.*, 1990). One of the observed side-effects was local irritation with redness and swelling at the IL-2 injection sites, which has been reported to be tolerable to the patients (Atzpodien *et al.*, 1990).

We observed a lobular panniculitis during sc treatment with IL-2, which was exacerbated during subsequent iv IL-2 administration.

A 40 year old woman with metastatic renal cell cancer was treated with $18 \times 10^6$ International Units (IU) IL-2/m$^2$/day (Eurocetus BV, Amsterdam) by sc injection for 5 days. The patient experienced pain at the injection sites on her left thigh following the last day of treatment. Three days thereafter she had to be admitted to hospital because of severe pain in the left leg, accompanied by redness, swelling, stiffness in the thigh and fever. It was thought that she might have a cellulitis of her left leg. Blood cultures were taken and the patient was treated with flucloxacillin. The blood cultures remained sterile. The redness, swelling and stiffness of the left leg gradually declined. Because the patient had also experienced rather severe local reactions at other IL-2 injection sites, it was decided to treat her further with an iv bolus schedule of $18 \times 10^6$ IU IL-2/m$^2$/day for 5 days after 3 weeks rest. During this cycle the pain at the former injection sites recurred especially on the left thigh. A biopsy was taken of this region, which showed small lymphocytic infiltrates around the blood vessels of the epidermis without vasculitis. In the subcutaneous fatty tissue an inflammatory process was observed, which consisted mainly of T cells, macrophages and eosinophils. A small percentage of T cells were CD25 positive ($\beta$ chain of the IL-2 receptor). The majority of the T cells were HLA-DR negative. The infiltrate contained equal numbers of CD4 and CD8 positive cells and was located in the lobuli of the fatty tissue. Fat necrosis was present in some places (Figure 1). The histology resembled that of the relapsing nodular non-suppurative panniculitis (Weber-Christian disease) (Lever & Schaumber-Lever, 1990). This disease is characterised by the appearance of crops of tender nodules and plaques in the subcutaneous fat, usually in association with mild fever (Lever & Schaumberg-Lever, 1990). The lower extremities are predominantly involved. The pathogenesis of the disease is unknown. An immune mechanism may be responsible, because high levels of circulating immune complexes have been recorded in these patients (Lever & Schaumberg-Lever, 1990). Our patient showed, however, no elevated immunoglobulins or circulating immune complexes. In addition, no antibodies against IL-2 could be detected. This patient shows that severe local reactions at IL-2 injection sites can occur during subcutaneous IL-2 treatment, which necessitates interruption of IL-2 treatment. Cutaneous toxicities due to intravenously administered IL-2 include macular erythema, pruritis and desquamation (Gaspari *et al.*, 1987). The histologic changes observed were non-specific, consisting of lymphoid cells surrounding blood vessels in the papillary dermis with fewer of these cells in the epidermis (Gaspari *et al.*, 1987).

Sporadic cases of IL-2 associated erythema nodosum, fatal pemphigus vulgaris (IL-2 + Interferon-$\beta$) and life threatening bullous skin lesions (IL-2, concomitant administration of antibiotics) have been reported (Weinstein *et al.*, 1987; Mier *et al.*, 1988; Ramseur *et al.*, 1989; Staunton *et al.*, 1991).

As far as we know, the complication reported herein has not yet been described in the literature. The pathogenesis needs to be further elucidated. The local production of cytokines, local stimulation of lymphocytes, macrophages and other antigen presenting cells might all play a contributary role.

**Figure 1** Skin biopsy showing infiltrates within the lobules of the subcutaneous fat (lobular panniculitis). Inset shows foam cells among the inflammatory infiltrate in which lymphocytes predominate.

Correspondence: J. Wagstaff, Free University Hospital, Department of Medical Oncology, De Boelelaan 1117, 1081 HV Amsterdam, Netherlands.
Received 17 March 1992.

**References**

ATZPODIEN, J., KÖRFER, A., FRANKS, C.R., POLIWODA, H. & KIRSCHNER, H. (1990). Home therapy with recombinant Interleukin-2 and Interferon-α 2b in advanced human malignancies. *Lancet*, **335**, 1509.

GASPARI, A.A., LOTZE, M.T., ROSENBERG, S.A., LOTZE, M.T., ROSENBERG, S.A., STEVEN, J.B. & KATZ, S.I. (1987). Dermatologic changes associated with Interleukin-2 administration. *JAMA*, **258**, 1624.

LEVER, W.F. & SCHAUMBERG-LEVER, G. (1990). Inflammatory diseases of the subcutaneous fat. In *Histopathology of the Skin*. Lever, W.F. & Schaumberg-Lever, G. (eds). J.B. Lippincott Company Philadelphia, pp. 275–276.

MIER, J.W., ARONSON, F.R., NUMEROF, R.P., VACHINO, G. & ATKINS, M.B. (1988). Toxicity of immunotherapy with Interleukin-2 and lymphokine-activated killer cells. *Path. Immunopathol. Res.*, **7**, 459.

RAMSEUR, W.L., RICHARDS, F. & DUGGAN, D.B. (1989). A case of fatal pemphigus vulgaris in association with beta interferon and Interleukin-2 therapy. *Cancer*, **63**, 2005.

STAUNTON, M.R., SCULLY, M.C., LE BOIT, P.E. & ARONSON, F.R. (1991). Life threatening bullous skin eruptions during Interleukin-2 therapy. *J. Natl Cancer Inst.*, **83**, 56.

WEINSTEIN, A., BUJAK, D., MITTELMAN, A. & DAVIDIAN, M. (1987). Erythema nodosum in a patient with renal cell carcinoma treated with Interleukin-2 and lymphokine-activated killer cells. *JAMA*, **258**, 3120.